# Supplementary material for: Relationship between Respiratory Rate, Oxygen Saturation, and Blood Test Results in Dogs with Chronic or Acute Respiratory Disease: A Retrospective Study
Source: Vet Sci. 2024 Jan 10;11(1):27. doi: 10.3390/vetsci11010027 (PMC10818868; doi:10.3390/vetsci11010027)
Supplement: Supplementary file 1 [file vetsci-11-00027-s001.zip › Supplementary Materials Table S1.pdf]

Table S1. Reference values for various tests used in this study.

Inspection item		Value	
Respiratory rate (/min)		< 40 breaths	
SpO_2 (%)		95–100	
CBC	Red blood cell (/μl)	5.5–8.5 × 10^6	
	Hematocrit (%)	37–55	
	Hemoglobin (g/dl)	12–18	
	White blood cell (/μl)	6000–17000	
	Band neutrophil (/μl)	0–300	
	Segmented neutrophil (/μl)	3000–11500	
	Lymphocyte (/μl)	1000–4800	
	Monocyte (/μl)	150–1350	
	Eosinophil (/μl)	100–1250	
	Platelet (/μl)	200–500 × 10^3	
Blood gas analysis		Vein	Artery
	pH	7.36–7.40	7.39–7.43
	PCO_2 (mmHg)	40.4–44.6	33.8–39.6
	HCO_3 (mmol/L)	23.2–26.6	21.3–25.1
	BE (mmol/L)	-1.8–1.8	-2.5–1.1
CRP (μg/mL)		< 1	

CRP, C-reactive protein; CBC, complete blood count; PCO_2, partial pressure of carbon dioxide; HCO_3, bicarbonate; BE, base excess
