# Supplementary material for: Relationship between Respiratory Rate, Oxygen Saturation, and Blood Test Results in Dogs with Chronic or Acute Respiratory Disease: A Retrospective Study
Source: Vet Sci. 2024 Jan 10;11(1):27. doi: 10.3390/vetsci11010027 (PMC10818868; doi:10.3390/vetsci11010027)
Supplement: Supplementary file 1 [file vetsci-11-00027-s001.zip › Supplementary Materials Table S2.pdf]

Table S2. Evaluation criteria for acid-base disorders in venous and arterial blood gas analysis.

		pH	PCO_2 (mmHg)	HCO_3 (mmol/L)	BE (mmol/L)
Vein					
Acidosis	Metabolic	< 7.36	< 40.4 or normal	< 23.2	< -1.8
	Respiratory	< 7.36	> 44.6	> 26.6 or normal	> 1.8 or normal
	Mixed	< 7.36	> 44.6	< 23.2	< -1.8
Alkalosis	Metabolic	> 7.40	> 44.6 or normal	> 26.6	> 1.8
	Respiratory	> 7.40	< 40.4	< 23.2 or normal	< -1.8 or normal
	Mixed	> 7.40	< 40.4	> 26.6	> 1.8
Artery					
Acidosis	Metabolic	< 7.39	< 33.8 or normal	< 21.3	< -2.5
	Respiratory	< 7.39	> 39.6	> 25.1 or normal	> 1.1 or normal
	Mixed	< 7.39	> 39.6	< 21.3	< -2.5
Alkalosis	Metabolic	> 7.43	> 39.6 or normal	> 25.1	> 1.1
	Respiratory	> 7.43	< 33.8	< 21.3 or normal	< -2.5 or normal
	Mixed	> 7.43	< 33.8	> 25.1	> 1.1

PCO_2, partial pressure of carbon dioxide; HCO_3, bicarbonate; BE, base excess
